# Xanthatin Induces Leishmanicidal Activity by Affecting Carbon Metabolism in Amastigotes 

**DOI:** 10.22037/ijpr.2021.114937.15122

**Published:** 2021

**Authors:** Ziba Akbari, KeyKavoos Seyfouri, Roghayeh Mirzazadeh, Elena Jamali, Zahra Zamani, Mohammad Arjmand

**Affiliations:** a *Metabolomics Laboratory. Pasteur Institute of Iran, Tehran, Iran. *; b *Department of Pathology, Loghman Hakim Hospital, Shahid Beheshti University of Medical Science, Tehran, Iran.*

**Keywords:** Xanthatin, Leishmania major, Metabolomics, Leishmania metabolome, 1HNMR Spectroscopy

## Abstract

Cutaneous leishmaniasis is caused by protozoa of the genus *Leishmania* and spread by sandflies. The standard therapy for this ailment is the first-line medication of pentavalent antimonial and the second drug line of pentamidine amphotericin B. All are practiced over the years and exhibit adverse toxicity effects. Herbal product-derived medicine is a promising potential source for treating parasitic diseases. Xanthatin, a xanthanolide sesquiterpene lactone, is isolated from *Xanthium strumarium* L. treats several ailments in many countries. In the present study, we investigated the leishmanicidal activity of the xanthatin by using a metabolomics-based analysis in J774 macrophages and amastigotes phases in *Leishmania major*. Xanthatin was isolated and identified by NMR spectroscopy. Macrophage toxicity of xanthatin performed by MTT assay. Macrophages infected by the *L. major’s* promastigote stationary phase, the infection rate (IR), and multiplication index (MI) were calculated. Axenic amastigotes were treated with xanthatin. Cell quenching and metabolite extraction were performed, and the metabolome profile was analyzed with NMR spectroscopy. Outliers were classified by using multivariate statistical analysis software, and relevant metabolites and pathways were worked out. The xanthatin IC_50_ rate defined 0.75 µg/mL base on macrophages viability and also *in-vitro* activity of xanthatin on amastigotes showed the best leishmanicidal activity in IR and MI values of 53% and 62.5%, respectively. Xanthatin altered amino sugars and nucleotide sugars metabolism, starch and sucrose metabolism, cyanoamino acid, and galactose metabolism. Our finding revealed that the main target of xanthatin is carbon metabolism, which is an essential step for amastigotes virulence.

## Introduction

Cutaneous leishmaniasis (CL) is considered a protozoan zoonotic disease induced by protozoa of the genus *Leishmania* and transmitted by sandflies to various mammal hosts, including humans. CL has an annual incidence of 0.6 to 1 million cases, with a mortality rate of 60000 per year (1). However, disease incidents are increasing worldwide nowadays. CL causes by over 17 *Leishmania* species, in which old-world *L major* species are the foremost causes of acute CL. CL therapy’s standard drug is the first-line drug of pentavalent antimonial (Sbv), meglumine antimoniate (glucantime), and the second drug line, pentamidine amphotericin B, which used for over the years, and they show adverse reactions and toxicity (2, 3).

Herbal product-derived medication is an encouraging potential source for treating parasitic diseases such as artemisinin, a sesquiterpenoid lactone compound in malaria. Xanthatin is also natural sesquiterpene lactones isolated from *Xanthium strumarium* L., an annual herb that belongs to the family *Asteraceae* genus *Xanthium * (4, 5). Sesquiterpenoid lactone, which is also called xanthanolides, is an active component of these medicinal plants and is traditionally used to remedy several ailments in many countries (6). Xanthatin with formula ‎C_15_H_18_O_3_ and molecular weight of ‎246.3 possesses remarkable anti-proliferative activity against various tumor cells *in-vitro* and *in-vivo* through downregulating the STAT3, GSK3B, and Beta-catenin (6-9). It also triggered ChK1-mediated DNA damage and destabilized Cdc25C via lysosomal degradation. Nibret* et al. *described the anti-inflammatory activity of xanthatin by inhibiting both PGE2 synthesis and 5-lipoxygenase activity (10). Xanthanolides possess antimicrobial, anti-protozoal, and cytotoxic activities (11). Lavault *et al.* described the leishmanicidal actions of xanthanolides isolated from *Xanthium macrocarpum* DC. They recorded that xanthinin was the most active xanthanolides toward L. *infantum* and L. *Mexicana*, with xanthatin against L. *infantum* (12).

The recent report on macrophages’ immune metabolism has shown metabolic repolarization as an attractive therapeutic target for disease conditions (13). The concept of unlike phenotypes of classically (M1) and alternatively (M2) activated macrophages was initially being proposed by Mills (14), and Thapa and Lee (15) explained its biochemical pathway signature variations. It postulated that macrophage’s metabolism could shift from an aerobic (with oxygen) profile based on oxidative phosphorylation to an anaerobic (without oxygen) one based on glycolysis and vice-versa (16). These metabolome profiles provide energy support for immune activity and directly affect immune cell functions by controlling transcriptional and post-transcriptional events in macrophages’ immunity, leading to destroying pathogens or being sacrificed (16).

Recent advances in metabolomics are opening up new horizons for understanding the metabolites signals that regulate “immunity” (17). Metabolomics is a new field of studying the metabolome profiling of a living organism with nuclear magnetic resonance spectroscopy. In a series of investigations, we consider the leishmanicidal activity of the various xanthanolides fraction of *X. Strumarium* in CL. The present study aimed to determine the effect of xanthatin on *L major* amastigotes and mice macrophage’s metabolism on *L. major’s* growth and viability in cell-free culture and macrophages amastigotes.

## Experimental

*Plant material*

*X. strumarium* leaves obtained from Kermanshah Province, Iran. A voucher specimen was deposited in the central herbarium of Tehran University, Tehran (number 48241). 

*Preparation of plant crude extracts*

The air-dried leaves of *X. strumarium* (4 kg) were macerated with an Ethanol-water solution (80%) at room temperature for 72 h. The resulting extract was mixed with active charcoal, centrifuged to isolate plant pigments. This mixture was filtrated and concentrated under reduced pressure in rotavapor at 45 °C to obtain a dark brown syrup (450 g) (18). 

*Isolation and characterization of xanthatin*

Xanthatin was isolated, as described by Nibret *et al.* (10). In brief, 450 g of the residue obtained was mixed with 200 g silica gel and dried at 40 °C. The mixture was placed onto the top of the silica gel column chromatography (50 × 3 cm). The fractions were collected manually every 30 min after adding 100% cyclohexane followed by cyclohexane: ethyl acetate 9:1 (V/V) to 100% ethyl acetate in a 10% stepwise ratio to raise the solvent polarity. Totally 38 tubes were collected, all tube’s content was analyzed by thin-layer chromatography (TLC) under ultraviolet (UV) light along with xanthatin standard (ChemFaces CAS No. 26791-73-1). Based on the TLC results profile, xanthatin fraction eluted after cyclohexane: ethyl acetate 1:9 ratio passed through the column as mobile phases. Pooled fractions (tubes 21-26) were recrystallized with methanol to obtained white needle crystal as xanthatin. This procedure was repeated to collect adequate xanthatin (15 mg). Xanthatin identity was confirmed by NMR (Bruker-500 MHz), and its purity was checked by Nibret’s method (10, 19).

*Cell culture*

A Blab/c mice macrophage cell line (J774) was cultured in RPMI 1640 medium (Atocel), containing 10% FBS (Gibco), Penicillin100 U/mL - Streptomycin 100 µg/mL (Sigma) as antibiotics at 37 °C with 5% CO_2_.

*Parasite culture*

The isolated amastigote of *L. major* (strain MRHO/IR/75/ER) from the lymph node of infected BALB/c mice was grown in M199 medium (Sigma, Germany) supplemented with 10% FBS, Penicillin100 U/mL - Streptomycin 100 µg/mL, two mM L-glutamine, 0.1 mM adenosine, 0.5 μg⁄mL hemins, and 40 mM HEPES at 23-26 ºC for 5-6 days. The stationary phase of promastigotes was centrifuged at 3000 rpm for 10 min at 4 °C and used to infect J774 macrophages (20).

*Preparation of xanthatin*

Ten and half milligram of xanthatin dissolved in 1 mL DMSO 1% and M199 medium (without FBS), sterilized by 0.22 μm micro-filters as stock solution and kept at 4 °C until use.

*Macrophage toxicity assay*

J774 macrophages (8 × 10^3^cell/well) were seeded in every well of 96-well culture plate (in triplicates) and incubated in RPMI 1640 supplemented with 10% FBS 37 ºC with 5% CO_2_ to attach. After 24 h, all wells’ medium was replenished with different xanthatin fractions (1050 to 0.007 µg/mL). The wells with no xanthatin (only cells and medium) and the wells without cells and xanthatin were considered negative control and blank sequentially. Also, Amphotericin B was used as positive control. The plate was incubated at 37 °C for 48 h. After adding 20 µL thiazolyl blue tetrazolium bromide (MTT) reagent (5 mg/mL in PBS) into each well, the plate was kept at 37 °C until the purple formazan crystals were visible under a microscope after 4 h. All medium was then discarded, and added 100 µL of DMSO into each well, including controls, shacked gently to dissolve formazan crystals and observe purple. Every well’s absorbance was recorded at 570 nm against 630 nm as a reference using a plate reading spectrophotometer. The formula Below determined the value of 50% inhibitory concentrations (IC_50_) and Percentage macrophage viability (Equation 1) (21):

$\mathrm{Macrophage viability}(\%)=\frac{\left(\mathrm{Average absorbance in duplicate drug wells}-\mathrm{average blank wells}\right)}{\mathrm{Average absorbance control wells}-\mathrm{average blank wells}}\times 100$ Equation 1.

*Macrophage infection*

6× 10^4^ cells of J774 macrophages seeded in 24-well culture plates equipped with 10 mm coverslips. The coverslips containing cells were incubated 24 h to adhere in the M199 medium at 37 °C with 5% CO_2_. Then, non-adherent cells were washed with phosphate-buffered saline (PBS) and infected with a stationary phase of *L. major* (10:1 parasite/macrophage) at 37 °C 5% CO_2_ for 4 h. Non-internalized promastigotes were washed with PBS (4 times), and the plate was incubated again in M199 medium supplemented with 2% FBS for 24 h (21, 22).

*Anti-amastigote assay*

Axenic amastigotes were treated in triplicates by various concentrations of xanthatin (353.75 to 0.0007 mg/mL) once. The three wells, amastigotes without any xanthatin, considered negative controls, and amphotericin B, was used as a positive control to compare the efficacy of xanthatin. All M199 medium and xanthatin concentrations were replenished every 24 h for three days. Then, wells were washed with PBS, fixed with methanol, and stained with 10% Giemsa. The infection rate (IR Equation 2) and multiplication index (MI, Equation 3) in each stained coverslip were determined microscopically and calculated. Using two Formulas Below (21, 22):

$\mathrm{IR}\left(\%\right)=\mathrm{Number of infected macrophages in 100 macrophages}$ Equation 2.

$\mathrm{MI}\left(\%\right)=\frac{\mathrm{Number of amastigotes in Test culture}/\mathrm{100 macrophages})}{\mathrm{Number of amastigotes in Negative control}/100\mathrm{macrophages}}\times 100$ Equation 3.

*Treatment of amastigotes with xanthatin*

The number of 4 × 10^7^ J774 macrophages (in each 175 cm cell culture flask) infected with 4 × 10^8 ^stationary promastigotes of *L.major* (5 replicates experimental and five replicates as negative control). All samples were incubated in an M199 medium containing 2% FBS at 37 °C with 5% CO_2_ for 24 h. Based on the xanthatin IC_50_ value, every experimental flask was treated with 1.575 µg/mL of xanthatin. The medium was replenished daily for three days in every flask. After five days, cells in each flask were washed once in PBS 1X (pH 7.4) gently, trypsinized, and transferred to 50 mL falcon tubes (21). 

*Cell quenching and metabolites extraction for NMR analysis*

All falcons were centrifuged at 4000 rpm at 4 °C for 15 min. Each pellet (containing 4 × 10^7^ amastigotes, approximately) was washed using ice-cold PBS (three times) to remove any impurities and residual media. The quenching process was done by tubes immersing into liquid nitrogen until freezing and thawing in an ice bath (0 °C) (23). The cell extraction process for ^1^HNMR spectroscopy was done on quenched cells in each tube. In brief, Ice-cold 1.8 M perchloric acid (200 µL/10^6^ cells) was added, followed by 5 min the sonication in an ice bath to disrupt the amastigotes membrane. All samples were kept in an ice bath for one hour to allow the precipitation of potassium perchlorate. After centrifugation at 12000 rpm at 4 °C for 10 min, the supernatant’s pH adjusted to 7.2, the samples were lyophilized and stored at −80 °C until future analysis (24). 

^1^ *HNMR Spectroscopy*

Lyophilized sample (n = 10) dissolved in 600 µL phosphate D_2_O buffer (100 mM, pH 7.2) containing 1 mM trimethylsilyl propionate (TSP) as a chemical shift reference (δ = 0 ppm) and two mM imidazole as pH indicator (δ = 5.50 to 8.80 ppm). Finally, samples were centrifuged at 18000 rpm for 15 min at 4 °C, and 500 µL supernatants were transferred into an NMR probe. 

NMR spectra of samples were obtained at 500.13 MHz for proton observation at 298K using a Bruker AV-500 NMR spectrometer. One-dimensional ^1^HNMR spectra were recorded using a 10-µs pulse, 0.1 s mixing time, 3.0 s relaxation delay, 6009.6 Hz spectral width, and 3000 transients with standard 1D NOESY pulse sequence to suppress the water peak (23, 24).

*Data Analysis*

^1^HNMR spectra were pre-processed by applying custom-written ProMatab (V.3.3) code in MATLAB (v.7.8.0.347) to convert spectra into a suitable format for multivariant analysis. Spectra binned into 0.005 ppm chemical shift, and water peak at 4.7 ppm eliminated. Data normalization and Pareto scaling were performed before data classification. A supervised Partial Least Square- Discriminate Analysis (PLS-DA) classification technique was used to identify notable outliers between experimental groups. Human Metabolome Database (HMDB) and LeishCyc databases were utilized to discover the corresponding metabolites to spectral outliers. Metabolic pathways worked out by using online MetaboAnalyst Ver. 3.0 (www.Metaboanalyst.ca) a metabolomics analysis software and SPSS to determine the p values.

## Results

*Isolation and identification of xanthatin*

**Figure 1:**
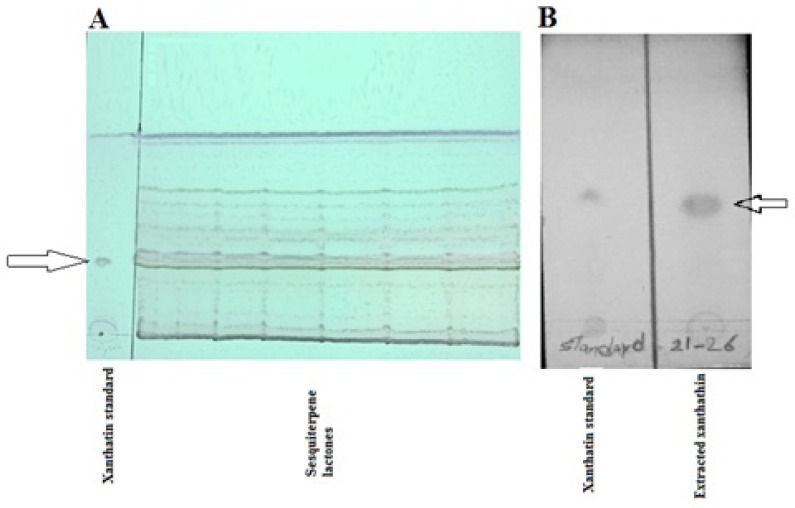
TLC preparative isolation of xanthatin fraction. (A) show the total sesquiterpene lactones fraction. (B) shows the isolated xanthatin

Adequate quantities of xanthatin (15 mg) were collected by column chromatography, followed by TLC techniques (Figure 1). Crystalized xanthatin was analyzed with NMR spectroscopy, and the spectra were in agreement with xanthatin NMR spectra provided along with standard by ChemFaces company and also Marco JA *et al.* (25) 

*Cytotoxicity effect of Xanthatin on Macrophages J774*

Cell viability results of different xanthatin concentrations on J774 macrophages are described in Table 1. Cell viability was raised with decreases in the stock extract concentration and xanthatin IC_50_ defined as 0.75 µg/mL. Nevertheless, xanthatin with our stock concentration (1050 µg/mL) showed the highest cytotoxic activity (100%).

The results for the activity of xanthatin and amphotericin B on amastigotes are depicted in Table 2. Based on xanthatin concentration (0.5 µg/mL), the rate of IR and MI of xanthatin was estimated at 53% and 62.5%, respectively, and this concentration was chosen for our further metabolomics NMR study.

*1H- NMR spectroscopy analysis*

**Figure 2:**
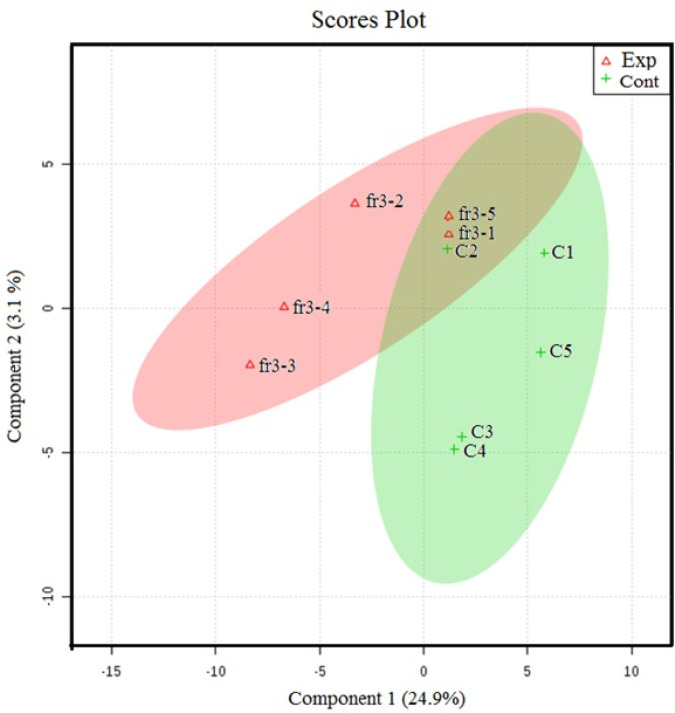
PLS-DA score plot classification between the selected PCs. The explained variances are shown in brackets

**Figure 3:**
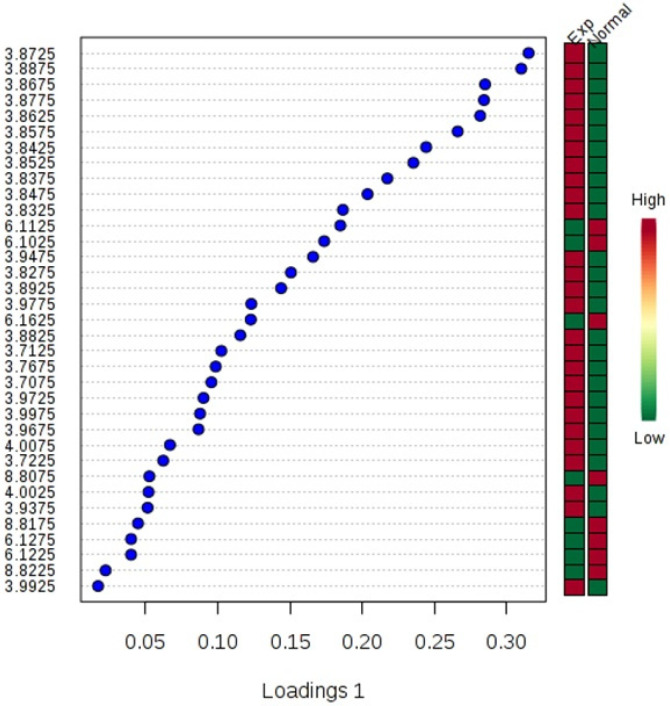
The variable important projection plot. The colored boxes on the right indicate the corresponding metabolite's relative concentrations in each group under study

**Figure 4:**
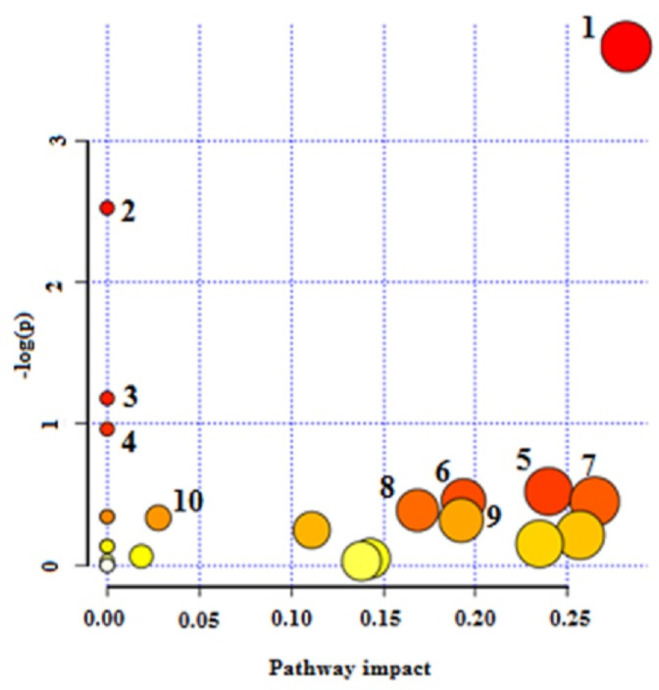
Pathway analysis degree of centrality. 1-Amino sugar and nucleotide sugar metabolism 2- Starch and sucrose metabolism 3- Cynoamino acid metabolism4- Galactose metabolism 5- Pentose phosphate pathway 6- Glycerolipid metabolism 7- Alanine, aspartate, and glutamate metabolism 8- Fructose and mannose metabolism 9- Pentose and glucuronate interconversions and 10- Valine, leucine, and isoleucine biosynthesis

The NMR spectroscopy analysis revealed a spectral peak variation in two groups of experiments; changes are more confined to regions 3, 6, and 8 ppm of spectra. PLS-DA score plot revealed that both groups had accurately classified (Figure 2), and the VIP plot explicated the chemical shift dimension (Figure 3). Most modified metabolites showed in Table 3, and the metabolic pathways analysis degree of centrality is described in Figure 4. The detail of the pathway’s analysis showed in Table 4. According to a statistical *p*-value of pathways value, less than 0.05 was taken for discussion in this article.

## Discussion

Pentavalent antimonial, *i.e*., amphotericin B, pentamidine, Miltefosine, and paromomycin, are known as efficient drug medications leishmaniasis therapy (26). Several mechanisms of action are proposed for these drugs; however, for a vast reason, like; host immune system, antileishmanial pharmacokinetics, and *leishmania* factors, their efficacy and drug resistance observed in leishmaniasis (27, 28).

**Figure 5:**
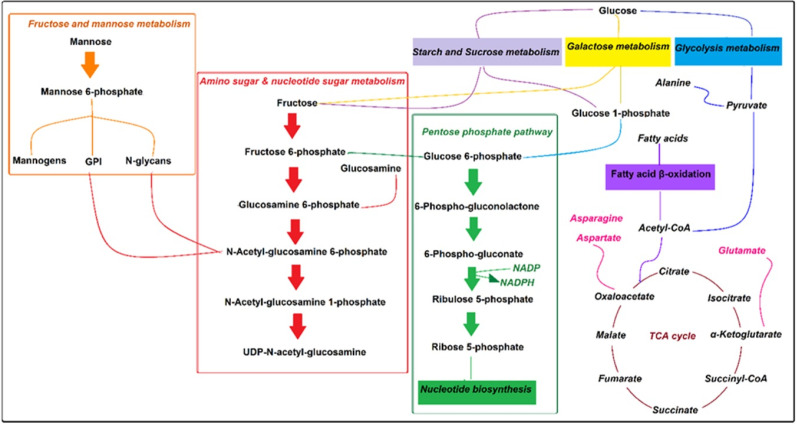
Pathway overview of the effect of xanthatin on amastigotes metabolome

Fatty acid uptake is the primary carbon source in amastigotes. Acetyl- CoA is produced by glycosomal pyruvate and fatty acid β-oxidation process required for the Krebs cycle to produce energy (29, 30). Therefore, the main drug target of pentavalent antimonies is based on the inhibition of *Leishmania* 𝛽-oxidation fatty acids and glycolysis pathway (26, 31). There are also records that the antileishmanial activity of Miltefosine is related to phospholipid biosynthesis lipid pathways and cytochrome C oxidase (26, 32). It is also stated that essential amino acids in amastigotes are usually utilized for protein synthesis (Figure 5), and paromomycin other antimony interferes with protein biosynthesis in the parasite (26, 27). 

Amphotericin B binds to membrane sterols and phospholipids and kills the parasite by altering membrane permeability, cellular potassium, magnesium, glucose, and water disbalance (26). Researchers declare that inferences including; up-regulation of glycolysis and the TCA cycle (33), changes in membrane fluidity and strolls composition, augmentation of membrane MDR1 pumps, and Drug Efflux, reducing reactive oxygen species and thiol, leading to drug amphotericin B resistance (26)*.* Based on our results, as the strength of anti leishmanicidal activity of drugs maters, xanthatin may show equal potency compared to amphotericin B, and the affected pathways are confined to amino sugar and nucleotide sugar metabolism, starch and sucrose metabolism, cyanoamino acid metabolism, Galactose metabolism, and pentose phosphate pathway only.

Generally, *Leishmania*’s energy demand is provided by carbon transformation in glycolysis, Krebs cycle, gluconeogenesis, and pentose phosphate pathway, which is called central carbon metabolism. It is believed that the living and prime proliferation of *Leishmania* amastigotes in mammalian hosts depend on phagolysosome metabolism.

Various types of sugars in phagolysosomes have emerged from the turnover of extracellular matrix compositions such as host glycoproteins, glycosoaminoglycans, and proteoglycans (34). Also, several genes related to hexosamines metabolism are reported in *Leishmania* genomes (35). 

Naderer *et al.* showed that scavenged hexosamines are vital sugars in *leishmania* phagolysosomes. Their catabolism is necessary to maintain critical biochemical pathways and prevent hexosamine toxicity (36). 

Malcolm J. *et al.* postulated that some phosphorylated hexosamines produce surface glycoconjugates like GPI and mannogens in glycosomal glycolysis and pentose phosphate pathways (30). So, these pathways play essential roles in the pathogenesis of amastigotes.

Based on statistical significance (*p*-value ≤ 0.05) within our findings, some hexosamines metabolites like; D-glucosamine 6-phosphate, N-Acetyl-alpha-D-glucosamine in amino sugar, and nucleotide sugar metabolism were the most changed metabolites. 

Amino sugars pathway in *L. major* amastigotes is utilized as sources of carbon and energy (36). Also, enough a concentration of amino sugars like glucosamine and N-acetyl glucosamine is needed to maintain the lowest hexosamines in the phagolysosome. They are transported to the glycosome and, after phosphorylation, convert to fructose-6-phosphate by some enzymes such as glucosamine 6-phosphate deaminase (GND).

Former results demonstrated that in a situation in which hexosamines are considered the primary source of carbohydrate, glycosomes of the *Leishmania *parasite could be targeted by GND, which plays an essential role in the growth and development of *leishmania* infection in susceptible animals (36).

 Fructose-6-phosphate is a crucial metabolite in glycoconjugate biosynthesis, and it is finally exported to the cytoplasm to produce compositions like mannogens and N-glycan. Our results showed that treated amastigotes by xanthatin exhibited some variations in hexose metabolites such as alpha-D-glucose, glucose 1-phosphate, beta-D-fructose, fructose 6-phosphate, and mannose 6-phosphate. 

Our results are then approved by earlier studies and suggest that any variation in the first metabolites of the amine sugar pathway or GND results in subsequent metabolite alterations of parasites. Moreover, our findings revealed that some metabolites, including; beta-D-fructose, alpha-D-glucose, and glucose 1-phosphate, have changed in starch and sucrose and Galactose metabolism pathways. Thus, it seems that the central role of xanthatin in amastigotes metabolism might have been performed by these critical metabolites, although further investigation should conduct to confirm this statement.

Some studies reported that the rate of amino acid oxidation elevates in *L.major* amastigotes. Amastigotes de novo pathway synthesizes non-essential amino acids like aspartate, alanine, and glutamate. On the contrary, with promastigotes, fatty acids have an essential role in *de novo* biosynthesis in phagolysosomes (34). Intracellular amastigotes are dependent on fatty acid β-oxidation and Krebs cycle for energy production. It is also stated that acetyl-CoA result from fatty acid β-oxidation is expensed in the Krebs cycle to produced intermediates for biosynthesis of non-essential amino acids, and they are used for biosynthesis of amino sugars, nucleotides, and thiol in amastigotes (36-38). The viability and virulence of amastigotes in phagolysosomes effect via disruption of key enzymes in the Krebs cycle for non-essential amino acid biosynthesis (29), because of the inhibition of some genes and proteins involved in β-oxidation and mitochondrion respiratory chain (39, 40). Our metabolomics-based study also revealed that the amastigote concentration of aspartate and asparagine amino acids in cyanoamino acid metabolism has changed as two targets for xanthatin. 

 Glucose 6-phosphate and 6-phosphogluconate in the pentose phosphate pathway are catalyzed by oxidative branch enzymes, including glucose-6-phosphate dehydrogenase, 6-phosphogluconate dehydrogenase to produced Ribose 5-phosphates and NADPH. Ribose 5-phosphates needed for DNA and RNA, another nucleotide biosynthesis (41), and NADPH plays a crucial role in antioxidant defenses against *Leishmania* species. Several studies focused on the pentose phosphate pathway role and its enzymes in some trypanosomes and *Leishmania* species ( (42, 43). Mehlotra *et al.* declared that there are not enough antioxidant enzymes available in *Leishmania* promastigotes, so they are sensitive to oxidative stress (44), and Deschacht focused on the relationship between oxidative stress mechanism and *Leishmania* infection (45). Moreover, Mukherjee *et al*. postulated that amphotericin B could affect macrophages’ antioxidant system (46, 47). Therefore, some researchers concluded that the pentose phosphate pathway could be considered as drug target for the treatment of leishmaniasis (43, 48). Our research revealed that Beta-D-glucose 6-phosphate, 6-D-phosphogluconate, and D-ribose 5-phosphate in the pentose phosphate pathway varied significantly between the two groups of study. Hence, our finding is commonly in line with prior reports.

**Table 1:** *In-vitro* activity of xanthatin on Macrophages

**Concentrations. (** µ **g/mL)**	**Xanthatin IC**_50_	**Amphotericin B. IC**_50_
1050	4.3 %	0%
525	5.1 %	0.1%
105	7.0%	2.1%
52.5	8.8%	2.3%
10.5	10.2%	8.6%
5.25	24.3%	19.2%
1.5	34.5%	28.8%
0.75	53.7%	49.3%
0.15	57.6%	51.4%
0.075	66.4%	65.9%
0.015	70.4%	76.5%
0.0075	75.2%	82.3%

**Table 2:** The activity of xanthatin on amastigotes

**Multiplication rate (%)**	**Infection rate (%)**	**Xanthatin Concentration (µg/mL)**
0%	0%	1050
10.2%	9%	5
23.5%	20%	3
48.0%	41 %	1
62.5%	53%	0.5
71.4%	61%	0.2
74.1%	63%	0.1
88.2%	75%	0.05
100%	90%	0.02
100%	85.3%	Negative control

**Table 3:** Metabolites affected by xanthatin

**Metabolite name**	**Metabolite name**	**Metabolite name**	**Metabolite name**
1,3-Butanediol	1,5-Anhydrosorbitol	1-Methylguanosine	D-Aspartic acid
2,3-Butanediol	2,4,5 Trimethoxybenzaldehyde	2-Methylbutyl acetate,	dCMP
3 Stachyose	3-Hydroxydodecanedioic acid	3-Methoxy-4-Hydroxyphenylglycol sulfate	Dehydroascorbic acid
3-Hydroxysebacic acid	3-Hydroxytetradecanedioic acid	3-Hydroxydodecanoic acid	Deoxyadenosine
3-Methoxytyrosine	3-Nitrotyrosine	3-Phosphoglyceric acid	Deoxyinosine
6-Phosphogluconic acid	7-Methylguanosine	Adenosine	Deoxyuridine
Adenosine monophosphate	Allocystathionine	Allose	Dethiobiotin
Alpha-D-Glucose	Alpha-Lactose	Atenolol	D-Fructose 2,6-bisphosphate
Azacitidine	Beta-D-Glucose 6-phosphate	Beta-N-Acetylglucosamine	D-Fructose
Cellobiose	Chlorogenic acid	Cytidine monophosphate	D-Galactose
Cytidine monophosphate	D-Glucose	Diethylthiophosphate	DL-Homocysteine
DL-O-Phosphoserine	D-Maltose	D-Mannose	D-Ribose 5-phosphate
D-Serine	D-Tagatose	Enilconazole	Ethenodeoxyadenosine
Flavin Mononucleotide	Fructose 6-phosphate	Galabiose	Galactonic acid
Galactose 1-phosphate	Galacturonic acid	Gluconolactone	Glucosamine 6-phosphate
Glucosamine 6-sulfate	Glucose 1-phosphate	Glucose 6-phosphate	Glyceric acid
Glycerol 3-phosphate	Glycerophosphocholine	Guaifenesin	Homocysteine
Hydroxypropionic acid	Inosine	Isomaltose	Isopropyl alcohol
Isopropyl alcohol	Isovalerylcarnitine	L-Alpha-aminobutyric acid	L-Arabinose
L-Arabitol	L-Asparagine	L-Aspartic acid	L-Cystathionine
L-Cystine	L-Gulonolactone	L-Hexanoylcarnitine	L-Histidine
L-Histidinol	L-Homocysteine	L-Homoserine	L-Iditol
L-Leucine	L-Octanoylcarnitine	L-Palmitoylcarnitine	L-Serine
L-Sorbose	Maltitol	Maltotetraose	Maltotriose
Mannitol	Mannose 6-phosphate	Melibiose	Methionine sulfoxide
Muramic acid	N-Acetylgalactosamine 4-sulphate	N-Acetylgalactosamine	N-Acetyllactosamine
N-Acetylmannosamine	N-Acetylneuraminic acid	N-Acetylserine	Neopterin
O-Phosphoethanolamine	Orotidine	Pseudouridine	Quinic acid
Uridine diphosphate-N-acetylglucosamine	Riboflavin	Ribonolactone	Sedoheptulose
Shikimic acid	Sorbitol	Sucrose	Thiamine
Threonic acid	Thymidine	Trehalose	Uridine 5'-monophosphate

**Table 4:** Pathway analysis detail

**Pathway**	**Total**	**Hits**	**-Log (p)**	**FDR**	**Metabolites**
Amino sugar and nucleotide sugar metabolism	21	8	0.00257	1.00	-D-Glucose 1-phosphate; -α-D-glucose; D-Mannose; Fructose 6-phosphate; D-Glucosamine 6 phosphate: -beta-D-fructose: -Mannose 6-phosphate : α-D-Glucosamine: UDP-N-Acetyl-alpha-D-glucosamine
Starch and sucrose Metabolism	6	3	0.00803	1.00	α-D-glucose; beta-D-fructose; D-Glucose 1-phosphate
Cynoamino acid metabolism	6	2	0.0308	1.00	L-aspartate; asparagine -
Galactose metabolism	7	2	0.0593	1.00	α-D-glucose; D-Glucose1-phosphate
Pentose phosphate pathway	16	3	0.0593	1.00	Beta-D-Glucose 6-phosphate; 6-D-phosphogluconate; D-Ribose 5-phosphate
Glycerolipid metabolism	11	2	0.0634	1.00	D-Glycerate; Glycerol 1-phosphate
Alanine, aspartate, and glutamate metabolism	17	3	0.0709	1.00	L-Aspartate; L-Asparagine; D-Glucosamine 6-phosphate
Fructose and mannose metabolism	18	3	0.0678	1.00	D-Mannose; D-Mannose 6-phosphate; Trimethylamine
Pentose and glucuronate interconversions	6	1	0.0709	1.00	D-Glucose 1-phosphate
Valine, leucine, and isoleucine biosynthesis	6	1	0.0709	1.00	L-Leucine

Total is the total number of compounds in the pathway; the Hits is the matched number from the user uploaded data; the p is the *p*-value; FDR is the false discovery rate; Metabolites are the altered metabolites in the pathway.

## Conclusion

Currently, there is a strong trend in herbal medicine for the treatment of leishmaniasis worldwide. To date, several research studies have focused on the therapeutic effects of *Xanthium* *Strumarium* extracts on leishmaniasis. This present pilot study was conducted for the first time to survey the antileishmanial activity of xanthatin and its impact on macrophages and amastigotes’ metabolism. Our finding suggests that the main target of xanthatin in amastigotes is carbon metabolism, which is an essential step for amastigotes virulence. Variations in the concentration of some essential metabolites in amino sugars and nucleotide sugars metabolism, starch and sucrose metabolism, cyanoamino acid, and galactose metabolism pathways might be considered valid drug targets. Nevertheless, additional investigations need to elucidate the biochemical and molecular mechanisms of action of xanthatin.

## Conflict of interests

The authors have no conflict of interest to declare.
